# Giant Optical Activity of Quantum Dots, Rods, and Disks with Screw Dislocations

**DOI:** 10.1038/srep14712

**Published:** 2015-10-01

**Authors:** Anvar S. Baimuratov, Ivan D. Rukhlenko, Roman E. Noskov, Pavel Ginzburg, Yurii K. Gun’ko, Alexander V. Baranov, Anatoly V. Fedorov

**Affiliations:** 1 ITMO University, 197101 Saint Petersburg, Russia; 2 Monash University, Clayton Campus, Victoria 3800, Australia; 3 Max Planck Institute for the Science of Light, Günther-Scharowsky-Straße 1, Bau 24, 91058 Erlangen, Germany; 4 Electrical Engineering Department, Tel Aviv University, Ramat Aviv 69978, Israel; 5 School of Chemistry and CRANN Institute, Trinity College, Dublin, Dublin 2, Ireland

## Abstract

For centuries mankind has been modifying the optical properties of materials: first, by elaborating the geometry and composition of structures made of materials found in nature, later by structuring the existing materials at a scale smaller than the operating wavelength. Here we suggest an original approach to introduce optical activity in nanostructured materials, by theoretically demonstrating that conventional achiral semiconducting nanocrystals become optically active in the presence of screw dislocations, which can naturally develop during the nanocrystal growth. We show the new properties to emerge due to the dislocation-induced distortion of the crystal lattice and the associated alteration of the nanocrystal’s electronic subsystem, which essentially modifies its interaction with external optical fields. The g-factors of intraband transitions in our nanocrystals are found comparable with dissymmetry factors of chiral plasmonic complexes, and exceeding the typical g-factors of chiral molecules by a factor of 1000. Optically active semiconducting nanocrystals—with chiral properties controllable by the nanocrystal dimensions, morphology, composition and blending ratio—will greatly benefit chemistry, biology and medicine by advancing enantiomeric recognition, sensing and resolution of chiral molecules.

Design and fabrication of new materials with superior optical properties—tailored for the execution of specific functions in purpose-built photonics devices—is a thriving area of materials science and engineering^1^. Traditional approaches to the development of new optical functionalities, based on changing the composition and geometry of structures made of naturally occurring materials, have been extensively exploited over last decades and are currently reaching their limits^2^,^3^,^4^,^5^,^6^,^7^. As a consequence, modern challenges faced by scientists and engineers are more and more tackled using new concepts of materials engineering, whose emergence was made possible by the considerable recent progress in nanotechnology. One of them is the nanostructuring of existing materials to produce *metamaterials* with optical properties beyond those available in nature^8^,^9^,^10^,^11^. This concept has already resulted in artificial materials exhibiting such counterintuitive phenomena as negative refraction and super-resolution^12^,^13^,^14^,^15^. A unique feature of metamaterial properties is their high tunability due to the possibilities of engineering the morphology of individual meta-atoms^16^,^17^ and variation the meta-atoms’ arrangement^13^,^18^,^19^,^20^.

A great deal of recent research efforts has been directed to chiral nanostructured materials, owing to their potential applications in chiral sensing and catalysis as well as in advanced photonics devices^21^,^22^,^23^,^24^. Such materials can be made of nanocrystals whose chirality originates from surface defects formed during the nanocrystal growth^25^,^26^,^27^,^28^,^29^,^30^,^31^. This paper develops a new concept of chiral semiconducting nanocrystals with bulk defects, which may serve as a uniform material base for the next-generation chiral selectors, markers and sensors. New chiral methods and devices based on such nanocrystals can potentially shape future pharmaceutical industry, natural-product chemistry, biochemistry and molecular biology^32^. As it happens in many branches of materials science, the advancement of optically active materials tends to occur through the replacement of “classical” molecules and crystals by predesigned nanostructures and their assemblies^21^. This approach provides multiple opportunities for the alteration of optical activity of such man-made materials. In particular, the frequency range of optical activity can be tuned by changing the dimensions of chiral nanostructures^33^,^34^,^35^ whereas the activity’s strength can be significantly enhanced by the nearby metallic nanoparticles^36^,^37^,^38^. Additional flexibility in the alteration of material activity comes from the freedom to arbitrarily arrange chiral or nonchiral meta-atoms in nonchiral or chiral superstructures^39^,^40^,^41^,^42^.

Here we pursue a promising avenue of creating artificial optical activity using the fact that quantum nanostructures are often formed with various kinds of defects, such as dislocations, disclinations, and impurities^43^,^44^,^45^,^46^,^47^,^48^,^49^,^50^. We propose an original way to introduce giant optical activity in semiconducting nanocrystals by creating screw dislocations inside them. This new concept is illustrated by a study of circular dichroism upon intraband transitions in three nanocrystals of the form of a circular cylinder: quantum dot, quantum rod, and quantum disk. The study points to the significance of the development of reliable production methods of enantiopure nanocrystals with bulk defects. Such a development will equip scientists and engineers with powerful tools of enantiomeric recognition and analysis, which will considerably advance the chiral methods of chemistry, biology and medicine.

## Quantum states of dislocation-distorted nanocrystals

**Figure 1:**
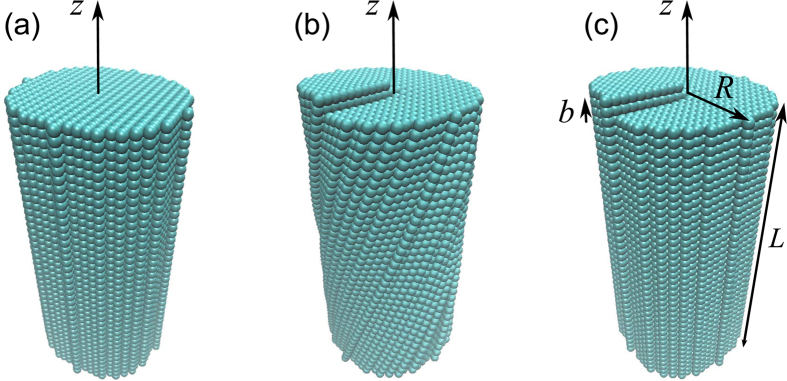
Cylindrical nanocrystals made of cubic semiconductor (a) without defects, (b) with a screw dislocation and Eshelby twist, and (c) with a screw dislocation and without Eshelby twist. Dislocation axis coincides with the cylinder axis *z* and *b* = 2*a*.

Let us consider a screw dislocation in a semiconducting nanocrystal with a simple cubic lattice (see Fig. 1). As was first noted by Eshelby^51^, the distortion of the crystal lattice by a screw dislocation creates torque, the partial relieving of which twists the nanocrystal as shown in Fig. 1(b). The aim of this work is to show that the residual internal strain and torsion of semiconducting nanocrystals with screw dislocations are among the major sources of their optical activity. The long-wavelength quantum states of electrons and holes confined by such nanocrystals are often described by assuming that the lattice distortion affects only the envelope wave functions whereas the Bloch amplitudes are the same as in the ideal lattice^52^,^53^. To enable the approximate analytical treatment of the nanocrystal’s electronic subsystem, we neglect the Eshelby twist and represent the semiconductor band structure by a pair of simple valence and conduction bands. Then the envelope wave functions inside the nanocrystal are the solution to the stationary Schrödinger equation in cylindrical coordinates^54^,^55^,^56^


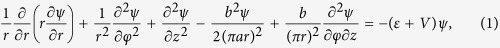


where *b* is the dislocation strength (Burgers vector projection onto the dislocation axis *z*), *a* is the undistorted-lattice constant, *ε* is the energy parameter, *V* is the confining potential, and where the last two terms on the left-hand side represent the elastic-deformation and kinetic parts of the dislocation Hamiltonian. Since this equation cannot be solved exactly even for the simplest nanocrystal in the shape of a cylinder, the kinetic potential 
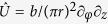
 will be treated using the first-order perturbation theory.

For the sake of convenience, we take the nanocrystal in the form of a circular cylinder and assume that its axis coincides with the axis of the dislocation, as shown in Fig. 1. We also assume that the nanocrystal’s surface is impenetrable for the confined carriers and require their wave functions to vanish at this surface. This assumption is justified for colloidal nanocrystals and nanocrystals hosted by wide band gap insulators^57^. The energies and wave function of the quantum states obeying Eq. (1) without the kinetic potential are given by


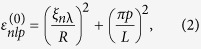



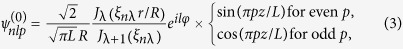


where *R* and *L* are the radius and length of the nanocrystal, 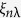 is the *n*th zero of the Bessel function of the first kind 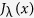, 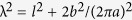, and 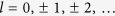 is the angular momentum projection. These expressions show that the elastic deformation blue shifts the quantum states of the dislocation-distorted nanocrystal with respect to the states of the dislocation-free nanocrystal (since 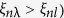, but does not discriminate between the states of opposite handednesses corresponding to opposite dislocation strengths ±*b*.

We next use this result to calculate the first-order corrections to the energies and wave functions of the nanocrystal states perturbed by the kinetic potential. Since this potential only couples the states of different parities (due to the presence of the *z* derivative), the first-order energy shift vanishes. The energies and wave functions of the perturbed quantum states are thus given by


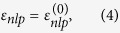



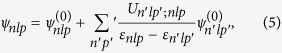


where the prime on the summation sign indicates that the summation is to be taken over the sets of quantum numbers 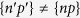,


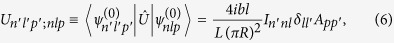



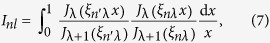


and where


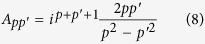


for *p* and 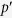 of different parities and 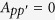 otherwise. Note that we have used the nondegenerate perturbation theory, because the kinetic potential does not couple the nanocrystal states with opposite projections of the angular momentum.

**Figure 2:**
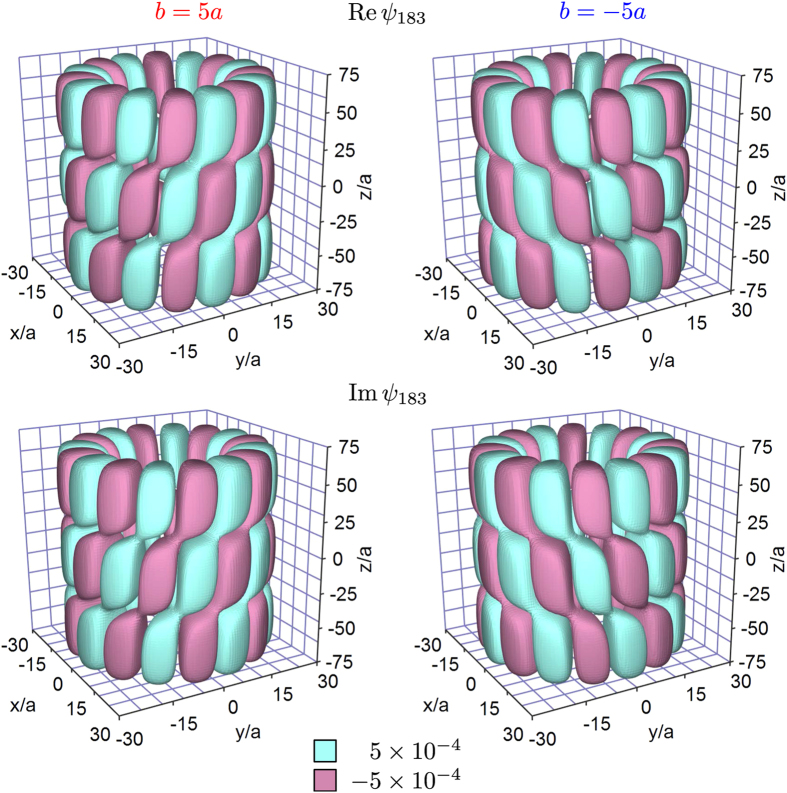
Chiral isosurfaces of real (upper panels) and imaginary (lower panels) parts of wave function *ψ*_183_ in cylindrical nanocrystals with right-handed (left panels) and left-handed (right panels) screw dislocations *b* = ±5*a*, *L* = 150*a*, and *R* = 30*a*.

The expressions presented in Eqs (2) and (3) through (6)–(8) and Fig. 2 provide the basis for the understanding of optical activity of semiconducting nanocrystals. They show that the quantum states of a dislocation-distorted nanocrystal possess a certain handedness, specified by the sign of the *z* component of the Burgers vector. Since this handedness controls the coherent superposition of the unperturbed nanocrystal states, it is reflected in all the electronic properties of the nanocrystal, including absorption and photoluminescence. The unperturbed states of different parities are seen to be coupled by 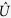 with strength that is determined by the product of the dislocation strength 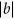 and the overlap integral 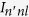. The absolute value of this integral grows with the quantum numbers 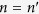 and 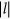, limiting the applicability of the perturbative approach. In what follows we shall focus on the transitions between the nanocrystal states that are not ‘accidently’ degenerate with respect to the quantum numbers *n* and *p*. One can show that the perturbation of state 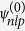 in this case is relatively small provided that 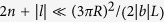. In a typical nanocrystal with *b* = *a*, *R* = 30*a*, and *L* = 50*a*, this condition is satisfied for more than 6000 quantum states, which significantly exceeds the total number of states in discrete energy spectra of real nanocrystals.

Noteworthy is that the kinetic potential in Eq. (1) is equivalent to the axially symmetric magnetic field 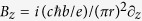 directed along the *z* axis. This effective magnetic field mixes up the states with the same projections of the angular momentum. To illustrate that these wave functions are essentially chiral, we plot in Fig. 2 the isosurfaces of their real and imaginary parts for *l* = 8 and relatively large dislocation strengths *b* = ±5*a*. As the figure suggests, the chirality of 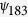 is determined by the chirality of the screw dislocation, i.e. by the sign of the dislocation strength.

### Circular dichroism

The handedness acquired by the electronic subsystem of a semiconducting nanocrystal in the presence of a screw dislocation manifests itself in different absorption rates of left-hand circularly polarized (LCP) and right-hand circularly polarized (RCP) light. The difference of these rates, referred to as circular dichroism (CD)^58^,^59^, is measurable upon both interband and intraband transitions of the confined charge carriers. To illustrate the effect of screw dislocation on the nanocrystal’s optical activity, we focus on intraband transitions of electrons. Without loss of generality, we consider a pump—probe experiment in which electrons are initially generated in certain quantum states of the conduction band through interband transitions caused by a linearly polarized optical pump. The energy of generated electrons is then increased further *via* intraband transitions, which are induced by a weak circularly polarized probe and used to measure the CD signal. All but the pump-excited states are assumed to be vacant whereas the depletion of the excited states is considered negligible. Taking into account the possible double degeneracy of the initial states with respect to the sign of the angular momentum projection and using the Fermi golden rule^60^, the CD signal can be written as


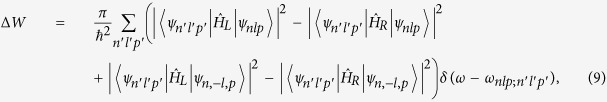


where 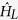 and 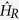 are the Hamiltonians describing the interaction of electrons with LCP and RCP light, 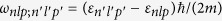 is the transition frequency, and we have assumed that the initial states of opposite momenta are populated with equal probabilities. The absolute values of the coefficients preceding the delta functions shall be referred to as the *CD strengths* of intraband transitions.

The presented expression demonstrates the importance of taking into account the retardation of the electric field over the nanocrystal volume^61^. If the effect of retardation is neglected, then an electron has equal probabilities of being excited from state 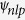 to state 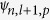 and from state 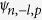 to state 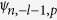 by circularly polarized photons of the same frequency but opposite handednesses. Mathematically, this reflects in the first term in Eq. (9) cancelling with the fourth term, and the second term cancelling with the third one.

By assuming that the probe is a plane electromagnetic wave propagating in the *z* direction and considering the exact spatial variation of its electric field over the nanocrystal’s length, we can write the Hamiltonian of the electron—photon interaction in the form^62^


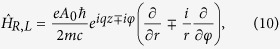


where −*e* and *m* are the charge and rest mass of a free electron, *A*_0_ is the amplitude of vector potential, *q* is the *z* component of the photon wave vector, and where the upper and lower signs correspond to RCP and LCP light, respectively. This Hamiltonian explicitly shows that the angular momentum of electron is increased by unity by LCP light and reduced by unity by RCP light. As a consequence, all the interband transitions can be split in pairs into three groups: (i) transitions from a state of zero angular momentum to states of momenta ±1, i.e. 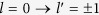; (ii) transitions 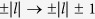; and (iii) transitions 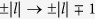. Note that owing to the double degeneracy of electronic states with 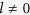, the energies of transitions in each group coincide.

The first nonvanishing contribution to the CD signal arises if one retains the first two terms in the Taylor series expansion of the spatial exponential *e*^*iqz*^. Using the perturbed wave functions given in Eq. (5) and the thus simplified electron—photon Hamiltonian, after some algebra (see Supplementary Information) we find


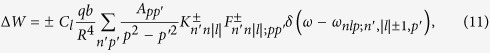


where the plus and minus signs correspond to the transitions of the first two groups and the third group, respectively, 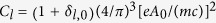, 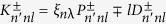,


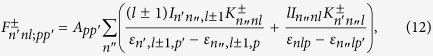



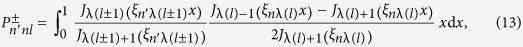


and


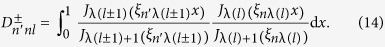


The obtained expressions show that the CD spectrum consists of resonant peaks associated with dipole-forbidden intraband transitions between unperturbed electronic states of different parities. The probability amplitudes of such transitions are given (up to constants) by the first two and the third compound matrix elements in Eq. (11). The product 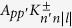 describes transitions between a pair of electronic states unperturbed by the kinetic potential, whereas the factors 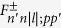 represent transitions between the unperturbed and perturbed states. The first and second terms in 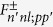 can be interpreted as proportional to the amplitudes of transitions from an unperturbed initial state 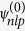 to a coherent superposition of final states 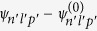, and from a superposition of initial states 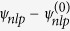 to an unperturbed final state 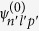, respectively.

The natural widths of spectral lines in a real CD spectrum of a semiconducting nanocrystal are inversely proportional to the total dephasing rates 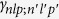 of the associated intraband transitions. To allow for the fact that the energy conservation law, expressed by the delta function in Eq. (11), is accurate to about 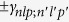, we replace the delta function by the Lorenzian


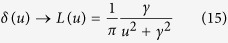


while assuming the natural widths of all spectral lines to be alike. Since the CD-active states decay *via* forbidden transitions, they have much longer lifetimes and much smaller total dephasing rates than the states decaying *via* electric dipole transitions at cryogenic temperatures, provided that nonradiative relaxation is relatively weak. This is because pure dephasing at low temperatures is weak^63^, and population relaxation of the dipole-allowed states is faster than that of the dipole-forbidden states in the absence of nonradiative decay^64^,^65^. Hence, in this case, the spectral lines in the CD spectrum are much sharper than those associated with the allowed transitions in the absorption spectrum. If the temperature of the system is high or strong nonradiative relaxation takes place for the CD-active states, then the two kinds of spectral lines may have comparable widths, dominated by the pure dephasing rates or by the natural linewidths, respectively.

### Optical activity of semiconducting nanocrystals

**Figure 3:**
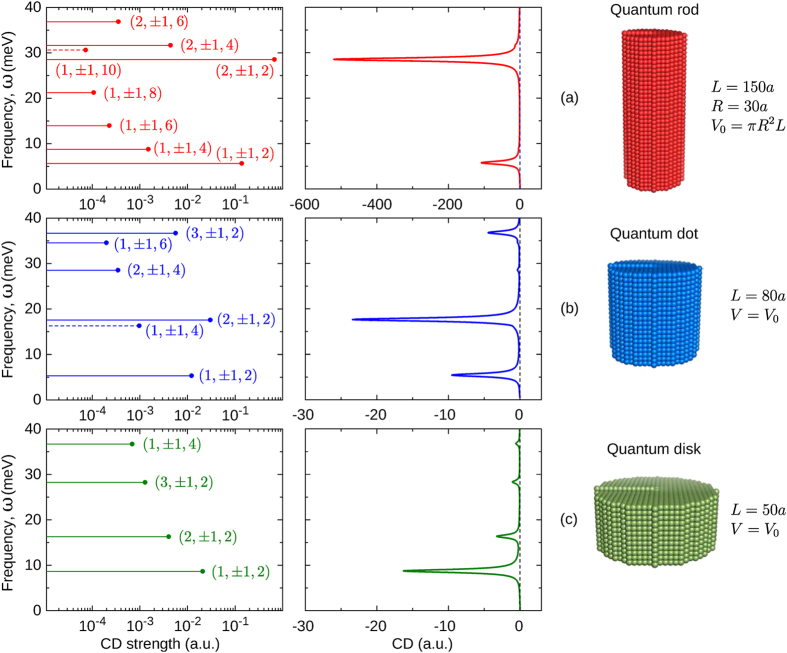
CD strengths (left panels) and CD spectra (right panels) of intraband transitions in ZnS nanocrystals. Pronounced CD is observed for (**a**) quantum dot, (**b**) quantum rod, and (**c**) quantum disk with right-handed screw dislocations *b* = *a*. All transitions occur from the ground state (101) of the nanocrystals and are represented by the spectral lines with FWHMs of 0.8 meV; negative and positive CD strengths are the solid and dashed lines, respectively; scaling factors are the same for all spectra. The volumes of the dot and disk are equal to the volume *V*_0_ of the rod.

Left panels in Fig. 3 show the CD strengths of intraband transitions in three equal-volume ZnS nanocrystals with right-hand screw dislocations *b* = *a*. The nanocrystals are referred to according to the ratio of their dimensions as: (a) quantum rod, with *L* = 150*a* and *R* = 30*a*; (b) quantum dot, with *L* = 80*a* and 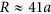; and (c) quantum disk, with *L* = 50*a* and 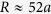. All the transitions occur from the ground state (*nlp*) = (101), appearing as resonant peaks in the CD spectra plotted on the right panels for *γ* = 0.4 meV^66^,^67^. The positions and intensities of lines in the spectra, as well as the signs of the CD strengths, are seen to be controllable by the nanocrystal dimensions. The general feature of all spectra is the dramatic decrease of the CD strengths with the difference of *p* and 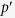 (as 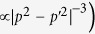, which makes transitions to states with 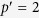 the most CD active for a given 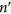 regardless of the nanocrystal dimensions. These transitions have the largest CD strengths in the quantum rod, appearing in its spectrum as sparse bands with a negligible CD signal between them. The CD bands of similar transitions in the quantum dot are about 10 to 100 times weaker but distributed over the spectrum more evenly, owing to the strict selection rules of the angular momentum. The quantum disk exhibits the weakest CD, which is the most pronounced upon the first intraband transition. Note that the CD strengths of all transitions but the two [to states (1, ±1, 10) of the quantum rod and (1, ±1, 4) of the quantum dot] are negative. These examples show that the effect of size quantization opens up wide opportunities for the engineering of optical activity of semiconducting nanocrystals as desired for their chiral applications.

**Figure 4:**
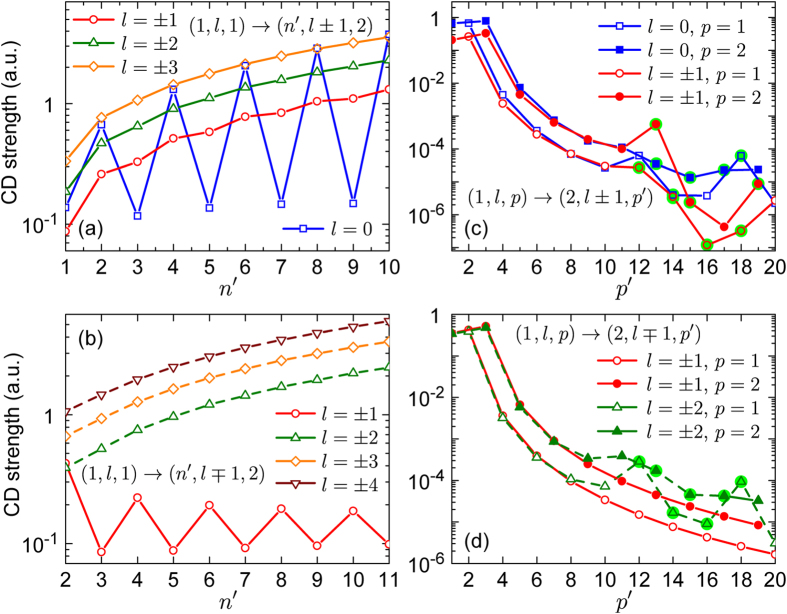
CD strengths of intraband transitions in ZnS quantum rod shown in Fig. 3. Transitions of the first and second groups are represented by (**a**,**c**), transitions of the third group are shown in (**b**,**d**). Solid and dashed guides for eyes mark negative and positive CD strengths, respectively. The signs of the CD strengths highlighted in green are opposite to the signs determined by the respective guides for eyes.

We next compare the CD strengths of the three groups of intraband transitions in the quantum rod. Variations of these strengths with quantum numbers of the final electronic states are shown in Fig. 4. Panels (a) and (b) show that transitions of the first two groups has negative CD strengths whereas the strengths of all the third-group transitions but the ones from state (1, ±1, 1) are positive. The oscillatory-type behavior of the CD strengths with varying parity of 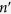 is peculiar to the transitions that are described solely by the first or second term in Eq. (12), with *l* = 0 or 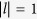, respectively. As descussed earlier, such transitions occur from or to electronic states unperturbed by the kinetic potential. The CD strengths of transitions between states with *n* = 1 and 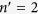 are shown in panels (c) and (d). Since the adopted classification of the transition groups is based on the selection rules of the angular momentum, the CD strengths of different groups similarly decay with the increase of 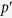. The strongest optical activity comes from transitions with 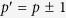, which is why there is one most active intraband transition (with 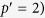 for *p* = 1, and a pair of such transitions (with 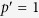 and 3) for *p* = 2. The irregular variations of the CD strengths at large 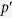 are due to the changes of the CD sign.

Figures 3 and 4 lead us to conclude that the maximum CD occurs upon intraband transitions of the second and third groups, from states with 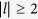, and upon a minimal change of the quantum number *p*. The weakest CD is exhibited upon transitions from or to the states of zero angular momentum, which are not modified by screw dislocations.

It is important to compare the obtained CD strengths with those exhibited by other chiral quantum objects. Since the strength of CD scales with the intensity of the probe, it is convenient to compare the respective dissymmetry factors, defined as the ratio of the CD strength to the sum of the absorption rates of LCP and RCP light, g = Δ*W*/*W*^58^,^68^. Table 1 shows the dissymmetry factors for several intraband transitions in ZnS quantum rod, quantum dot, and quantum disk. The values in the table exceed the typical dissymmetry factors of chiral molecules (10^−4^–10^−3^) by two to three orders of magnitude, and are comparable with giant g-factors of chiral plasmonic complexes (~0.3)^36^,^68^,^69^.

### Long nanocrystals

The analysis just presented is based on the expansion of the spatial exponential in the electron—photon Hamiltonian, which limits the applicability of Eqs. (11, 12, 13, 14) to the nanocrystals that are much smaller than the probe’s wavelength. This wavelength has to significantly exceed 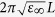 (where 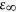 is the high-frequency permittivity of the nanocrystal), implying far infrared frequencies of intraband transitions often met in practice. When this is not the case, the CD spectrum of long nanocrystals can be calculated by keeping the full exponential to obtain (see Supplementary Information)


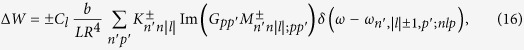


where, as before, ± signs correspond to the transitions of different groups,


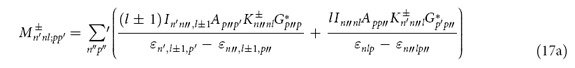


and


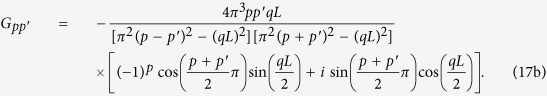


The prime on the summation sign in Eq. (17) indicates that the summation is to be taken over the sets of quantum numbers 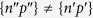 in the first term and 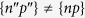 in the second term.

It is easy to show that Eq. (16) reduces to Eq. (11) in the case of short nanocrystals with *qL*  1, for which 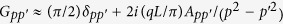. A further analysis of Eq. (16) shows that the CD signal weakens with the nanocrystal length as 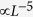. Since according to Eq. (11) the CD strength grows in short nanocrystals as 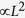, it must have a maximum at a certain nanocrystal length (strictly speaking, an increase of the nanocrystal length must be accompanied by the rise of the nanocrystal radius to retain the applicability of the perturbative expansion, but this fact does not change the conclusion). The wavelength of a typical intraband transition in Fig. 3 is of the order of hundreds of micrometers, so that the maximal CD strength would be achieved in nanocrystals longer than 10 *μ*m. This implies that the optical activity of a chiral semiconducting nanocrystal can be enhanced in practice by simply increasing the nanocrystal length.

As a concluding remark, it should be noted that the inhomogeneous size broadening in a CD-active nanocrystal ensemble can be taken into account by the standard averaging


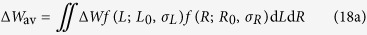


while assuming that the nanocrystal dimensions are distributed around their geometric means *L*_0_ and *R*_0_ lognormally, i.e.


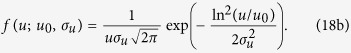


Taking into account the random orientation of nanocrystals in an ensemble is also quite straightforward. Some algebra shows that if the propagation direction of a circularly polarized probe makes an angle 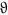 with the axis of a chiral nanocrystal, then the CD signal is proportional to 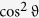. The averaging of this signal over all spatial orientations of the nanocrystals simply yields a factor of 1/2 (see Supplementary Information).

## Discussion

We have shown that screw dislocations make the host semiconducting nanocrystals essentially chiral and optically active. The optical activity results from the modification of the nanocrystal’s electronic subsystem caused by the topological distortion of its crystal lattice by the screw dislocation. To study this activity, we analytically calculated the CD signal upon intraband transitions in a dislocation-distorted nanocrystal, and analyzed its dependency on the nanocrystal’s dimensions and the quantum numbers of states involved in the transitions. The analysis revealed CD to appear upon forbidden transitions, grow with the nanocrystal length, and be directly proportional to the dislocation strength. More importantly, we showed that quantum dots, quantum rods, and quantum disks made of ordinary semiconducting materials may exhibit giant optical activity that significantly exceeds that of chiral molecules, and is comparable to the optical activity of chiral plasmonic complexes reported in the pivotal work of A. O. Govorov *et al.*^38^.

Since screw dislocations can naturally develop during the preparation of semiconducting nanocrystals, our study suggests that most of nanocrystals are likely to be inherently chiral and optically active. Unless special efforts are made to insure asymmetric fabrication, left-handed and right-handed dislocations are formed with equal probabilities, resulting in racemic mixtures of nanocrystals. It is therefore quite likely that many of optically inactive nanocrystals that have previously been reported in the literature^70^,^71^,^72^,^73^,^74^ were, in fact, racemic (50:50) mixtures of left-handed and right-handed enantiomers. The development of cheap and efficient methods of separation of nanocrystal enantiomers can potentially transform pharmaceutical industry, enabling recognition and sensing of chiral drug molecules and innovative racemate resolution methods. Since chirality plays key roles in chemical and biological systems, our findings are expected significantly impact the entire nanobiotechnology research. They may lead to the revision of currently existing knowledge and approaches in such crucial areas as nanotoxicology, and affect the application of nanoparticles in medical diagnostics and drug delivery. We are currently working on the realisation of the proposed pump-probe scheme, in order to experimentally test our theoretical findings. We are planning to measure and study the CD signal upon intraband transitions in our recently fabricated chiral nanocrystals made of CdSe and ZnS^75^. The results of this study will be published in our future paper elsewhere.

## Additional Information

**How to cite this article**: Baimuratov, A. S. *et al.* Giant Optical Activity of Quantum Dots, Rods, and Disks with Screw Dislocations. *Sci. Rep.* **5**, 14712; doi: 10.1038/srep14712 (2015).

## Supplementary Material

Supplementary Information

## Figures and Tables

**Table 1:** Dissymmetry factors for intraband transitions in three ZnS nanocrystals shown in Fig. 3.

Transition	Quantum rod	Quantum dot	Quantum disk
(101) → (1, ±1, 2)	−0.044	−0.068	−0.025
(101) → (1, ±1, 4)	−0.062	−0.051	−0.234
(101) → (2, ±1, 2)	−0.120	−0.151	−0.088
