# Supplementary material for: Giant Optical Activity of Quantum Dots, Rods, and Disks with Screw Dislocations
Source: Sci Rep. 2015 Oct 1;5:14712. doi: 10.1038/srep14712 (PMC4589690; doi:10.1038/srep14712)
Supplement: Supplementary Information [file srep14712-s1.pdf]

Supplementary Information for Paper

Giant Optical Activity of Quantum Dots, Rods, and Disks with Screw Dislocations

Anvar S. Baimuratov, Ivan D. Rukhlenko, Roman E. Noskov, Pavel Ginzburg, Yurii K. Gun'ko, Alexander V. Baranov, and Anatoly V. Fedorov

Screw dislocation in a semiconducting nanocrystal

Consider a screw dislocation along the axis of a cylindrical semiconducting nanocrystal of radius R and length L. This dislocation can be described by the scalar deformation potential $-2b^2/(2\pi a r)^2$ and the kinetic term $b/(\pi r)^2\partial_\varphi\partial_z$, where a is the lattice constant and b is the Burgers vector projection on the z axis.

The solution to the Schrödinger equation without the kinetic term

$$-\epsilon^{(0)}\psi^{(0)} = \left(\frac{1}{r}\frac{\partial}{\partial r}\left(r\frac{\partial}{\partial r}\right) + \frac{1}{r^2}\frac{\partial^2}{\partial\varphi^2} + \frac{\partial^2}{\partial z^2} - \frac{2b^2}{(2\pi a r)^2} + V(r,z)\right)\psi^{(0)}$$

and with the confinement potential of the form

$$V(r,z) = \begin{cases} 0 \ \text{ for } \ r \le R, |z| \le \dfrac{L}{2}, \\ \infty \ \text{ for } \ r > R, |z| > \dfrac{L}{2}, \end{cases}$$

is given by energies

$$\epsilon_{nlp}^{(0)} = (\xi_{n\lambda}/R)^2 + k_p^2$$

and wave functions

$$\psi_{nlp}^{(0)}(r,\varphi,z) = \frac{\sqrt{2}}{\sqrt{\pi L}R}\frac{J_\lambda(\xi_{n\lambda}r/R)}{J_{\lambda+1}(\xi_{n\lambda})}\cos(k_p z)e^{il\varphi}$$

for odd p and

$$\psi_{nlp}^{(0)}(r,\varphi,z) = \frac{\sqrt{2}}{\sqrt{\pi L}R}\frac{J_\lambda(\xi_{n\lambda}r/R)}{J_{\lambda+1}(\xi_{n\lambda})}\sin(k_p z)e^{il\varphi}$$

for even p. Here $J_\lambda(\xi_{n\lambda}) = 0$, $l = 0, \pm 1, \pm 2, \ldots$, $n = 1, 2, 3, \ldots$, $k_p = \pi p/L$, and

$$\lambda = \sqrt{l^2 + \frac{b^2}{2(\pi a)^2}}.$$

First-order perturbation theory for the kinetic term

The kinetic term $U = b/(\pi r)^2\partial_\varphi\partial_z$ can be treated as a small perturbation of the nanocrystal's electronic subsystem. Since the Bessel function order (λ) is nonzero for all l, the matrix elements of perturbation U are all finite and given by

$$U_{n'l'p';nlp} \equiv \left\langle \psi^{(0)}_{n'l'p'} \middle| U \middle| \psi^{(0)}_{nlp} \right\rangle = \frac{4ilb}{L(\pi R)^2} I_{n'nl} \delta_{ll'} A_{pp'},$$

where

$$I_{n'nl} = \int_0^1 \frac{J_\lambda(\xi_{n\lambda} u)}{J_{\lambda+1}(\xi_{n\lambda})} \frac{J_\lambda(\xi_{n'\lambda} u)}{J_{\lambda+1}(\xi_{n'\lambda})} \frac{du}{u},$$

$$A_{pp'} = \int_{-\frac{\pi}{2}}^{\frac{\pi}{2}} du \sin(p'u) \frac{d[\cos(pu)]}{du} = \int_{-\frac{\pi}{2}}^{\frac{\pi}{2}} du \cos(p'u) \frac{d[\sin(pu)]}{du} = (-1)^{\frac{p+p'+1}{2}} \frac{2pp'}{p^2 - p'^2}$$

if p and p' have different parities, and $A_{pp'} = 0$ if p and p' are of the same parity.

The first-order energies and wave functions of the nanocrystal electrons are of the form

$$\epsilon_{nlp} = \epsilon^{(0)}_{nlp},$$

$$\psi_{nlp} = \psi^{(0)}_{nlp} + \sideset{}{'}\sum_{n_1 p_1} \frac{U_{n_1 l p_1; nlp}}{\epsilon_{nlp} - \epsilon_{n_1 l p_1}} \psi^{(0)}_{n_1 l p_1}.$$

The energy correction is zero because $U_{nlp;nlp} \propto A_{pp} = 0$.

Applicability limits of the perturbation theory

Consider the smallness parameter

$$\alpha = \left| \frac{U_{n'lp';nlp}}{\epsilon_{nlp} - \epsilon_{n'lp'}} \right|.$$

Since p is the only quantum number always changed by the perturbation, the denominator of α can have either four or two terms, depending on whether the principal quantum number n is changed or not:

$$\epsilon_{nlp} - \epsilon_{n'lp'} = \frac{\left(\xi^2_{n\lambda} - \xi^2_{n'\lambda}\right)}{R^2} + \frac{\pi^2}{L^2}\left(p^2 - p'^2\right)$$

or

$$\epsilon_{nlp} - \epsilon_{nlp'} = \frac{\pi^2}{L^2}\left(p^2 - p'^2\right).$$

Let us first assume that the principal quantum number does not change. Then

$$\alpha = \left| \frac{U_{nlp';nlp}}{\epsilon_{nlp} - \epsilon_{nlp'}} \right| = \left| \frac{4lbLI_{nnl}A_{pp'}}{\pi^4 R^2 \left(p^2 - p'^2\right)} \right|.$$

Since

$$I_{nnl} = \int_0^1 \left(\frac{J_\lambda(\xi_{n\lambda} u)}{J_{\lambda+1}(\xi_{n\lambda})} \right)^2 \frac{du}{u} = \frac{\xi_{n\lambda}}{2\lambda} \frac{1}{J_{\lambda+1}(\xi_{n\lambda})} \frac{\partial J_\lambda(u)}{\partial \lambda} \bigg|_{u=\xi_{n\lambda}},$$

$$\left| \frac{A_{pp'}}{p^2 - p'^2} \right| \leq \frac{A_{21}}{3} = \frac{4}{9},$$

and $\lambda > |l|$, then

$$\alpha \leq \frac{8|b|L}{9\pi^4 R^2} \frac{\xi_{n\lambda}}{|J_{\lambda+1}(\xi_{n\lambda})|} \left| \frac{\partial J_\lambda(u)}{\partial \lambda} \bigg|_{u=\xi_{n\lambda}} \right|.$$

For $b \sim a$ we have $\xi_{n\lambda} \approx \xi_{n|l|}$, $\lambda \approx |l|$, and

$$\left| \frac{\partial J_\lambda(u)}{\partial \lambda} \bigg|_{u=\xi_{n\lambda}} \right| \lesssim \left| \frac{\partial J_l(u)}{\partial l} \bigg|_{u=\xi_{n|l|}} \right| \lesssim \frac{\pi}{2} \left| Y_{|l|}(\xi_{n|l|}) \right| = \frac{1}{\xi_{n|l|} \left| J_{|l|+1}(\xi_{n|l|}) \right|}.$$

This leads us to an estimate

$$\alpha \leq \frac{8|b|L}{9\pi^4 R^2} \frac{1}{\left[J_{|l|+1}(\xi_{n|l|}) \right]^2}.$$

We then can write

$$\left[J_{|l|+1}(\xi_{n|l|}) \right]^2 \sim \frac{2}{\pi \xi_{n|l|}} \sim \frac{2}{\pi^2 (n + |l|/2 - 1/4)} \gtrsim \frac{4}{\pi^2 (2n + |l|)},$$

and

$$\alpha \lesssim \frac{2(2n + |l|)}{9\pi^2} \frac{|b|L}{R^2}.$$

In the general case of changing n, the smallness parameter is of the form

$$\alpha = \left| \frac{4lbI_{n'nl}A_{pp'}}{\pi^2 L \left[\left(\xi_{n\lambda}^2 - \xi_{n'\lambda}^2 \right) + \pi^2 \left(p^2 - p'^2 \right) (L/R)^2 \right]} \right|.$$

Since

$$I_{n'nl} < \frac{I_{n'n'l} + I_{nnl}}{2},$$

$$\xi_{n\lambda} \sim \xi_{n|l|} \sim \pi \left(n + \frac{|l|}{2} \right),$$

and

$$\left| A_{pp'} \right| \leq A_{21} = \frac{4}{3},$$

we have

$$\alpha \lesssim \frac{2}{3\pi^2} \frac{n + n' + |l|}{\left| (n - n')(n + n' + |l|) + \left(p^2 - p'^2 \right) (R/L)^2 \right|} \frac{|b|}{L}.$$

If $n = n'$, we recover the previous result

$$\alpha \lesssim \frac{2(2n + |l|)}{9\pi^2} \frac{|b|L}{R^2},$$

which shows that state (nlp) is well described by the first-order perturbation theory, provided that

$$2n+|l| \ll \frac{(3\pi R)^2}{2|b|L}.$$

For $n' < n$ and $p' < p$ we have

$$\alpha \lesssim \frac{2}{3\pi^2}\frac{n+n'+|l|}{n+n'+|l|+(2p-1)(R/L)^2}\frac{|b|}{L} < \frac{2}{3\pi^2}\frac{|b|}{L}.$$

For $n' > n$ and $p' > p$ we have

$$\alpha \lesssim \frac{2}{3\pi^2}\frac{n+n'+|l|}{n+n'+|l|+(2p'-1)(R/L)^2}\frac{|b|}{L} < \frac{2}{3\pi^2}\frac{|b|}{L}.$$

These conditions are satisfied automatically, as $3\pi^2 L \gg 2|b|$ for all real nanocrystals with screw dislocations.

For $n' > n$ and $p' < p$, as well as for $n' < n$ и $p' > p$, we have

$$\alpha \lesssim \frac{2}{3\pi^2}\frac{n+n'+|l|}{\left||n-n'|(n+n'+|l|)-|p-p'|(p+p')(R/L)^2\right|}\frac{|b|}{L}.$$

The denominator of this expression may be small only in case of accidental degeneracy of electronic states, i.e. if the energies of states with different quantum numbers n, n', p and p' coincide. In this paper, we assume that such an accidental degeneracy is absent.

Interaction of electrons with circularly polarized light

To study intraband transitions in our nanocrystal, we employ the **Ap** representation of light–matter interaction. Consider left-hand and right-hand circularly polarized plane waves of wave vector $q=\sqrt{\varepsilon}\omega/c$ propagating along the z axis. The interaction of electrons with such waves can be represented by operators

$$H_R = -i\frac{A_0 e}{\sqrt{2}mc}e^{iqz}p_{+1} \approx \frac{A_0 e\hbar}{2mc}(1+iqz)e^{-i\varphi}\left(\frac{\partial}{\partial r}-\frac{i}{r}\frac{\partial}{\partial\varphi}\right) = \frac{A_0 e\hbar}{2mc}Q_+,$$

$$H_L = i\frac{A_0 e}{\sqrt{2}mc}e^{iqz}p_{-1} \approx \frac{A_0 e\hbar}{2mc}(1+iqz)e^{i\varphi}\left(\frac{\partial}{\partial r}+\frac{i}{r}\frac{\partial}{\partial\varphi}\right) = \frac{A_0 e\hbar}{2mc}Q_-.$$

The matrix elements of implicitly defined operators $Q_\pm$ upon transitions occurring without a change of the parity of quantum number p are given by

$$\langle\psi_{n'l'p'}|Q_\pm|\psi_{nlp}\rangle = \left\langle\psi^{(0)}_{n'l'p'}\middle|e^{\mp i\varphi}\left(\frac{\partial}{\partial r}\mp\frac{i}{r}\frac{\partial}{\partial\varphi}\right)\middle|\psi^{(0)}_{nlp}\right\rangle$$

whereas the matrix elements upon transitions with the parity change are:

$$\langle\psi_{n'l'p'}|Q_\pm|\psi_{nlp}\rangle$$

$$= \left\langle\psi^{(0)}_{n'l'p'}\middle|iqze^{\mp i\varphi}\left(\frac{\partial}{\partial r}\mp\frac{i}{r}\frac{\partial}{\partial\varphi}\right)\middle|\psi^{(0)}_{nlp}\right\rangle$$

$$+\sum_{n_1p_1}{}^{'}\frac{U_{n'l'p';n_1l'p_1}}{\epsilon_{n'l'p'}-\epsilon_{n_1l'p_1}}\left\langle\psi^{(0)}_{n_1l'p_1}\middle|e^{\mp i\varphi}\left(\frac{\partial}{\partial r}\mp\frac{i}{r}\frac{\partial}{\partial\varphi}\right)\middle|\psi^{(0)}_{nlp}\right\rangle$$

$$+\sum_{n_1p_1}{}^{'}\frac{U_{n_1lp_1;nlp}}{\epsilon_{nlp}-\epsilon_{n_1lp_1}}\left\langle\psi^{(0)}_{n'l'p'}\middle|e^{\mp i\varphi}\left(\frac{\partial}{\partial r}\mp\frac{i}{r}\frac{\partial}{\partial\varphi}\right)\middle|\psi^{(0)}_{n_1lp_1}\right\rangle.$$

It can be shown that

$$\left\langle\psi^{(0)}_{n'l'p'}\middle|ze^{\mp i\varphi}\left(\frac{\partial}{\partial r}\mp\frac{i}{r}\frac{\partial}{\partial\varphi}\right)\middle|\psi^{(0)}_{nlp}\right\rangle=\frac{4L}{\pi^2R}K^{\mp}_{n'nl}\delta_{l',l\mp1}B_{pp'},$$

$$\left\langle\psi^{(0)}_{n'l'p'}\middle|e^{\mp i\varphi}\left(\frac{\partial}{\partial r}\mp\frac{i}{r}\frac{\partial}{\partial\varphi}\right)\middle|\psi^{(0)}_{nlp}\right\rangle=\frac{2}{R}K^{\mp}_{n'nl}\delta_{l',l\mp1}\delta_{pp'},$$

where

$$K^{\mp}_{n'nl}=\xi_{n\lambda}P^{\mp}_{n'nl}\pm lD^{\mp}_{n'nl},$$

$$B_{pp'}=\int_{-\frac{\pi}{2}}^{\frac{\pi}{2}}udu\sin(p'u)\cos(pu)=\int_{-\frac{\pi}{2}}^{\frac{\pi}{2}}udu\cos(p'u)\sin(pu)=\frac{2A_{pp'}}{p^2-p'^2}$$

(here p' and p have different parity)

$$D^{\pm}_{n'nl}=\int_0^1\frac{J_{\lambda(l)}(\xi_{n\lambda(l)}u)}{J_{\lambda(l)+1}(\xi_{n\lambda(l)})}\frac{J_{\lambda(l\pm1)}(\xi_{n'\lambda(l\pm1)}u)}{J_{\lambda(l\pm1)+1}(\xi_{n'\lambda(l\pm1)})}du,$$

and

$$P^{\pm}_{n'nl}=\int_0^1\frac{J_{\lambda(l)-1}(\xi_{n\lambda(l)}u)-J_{\lambda(l)+1}(\xi_{n\lambda(l)}u)}{2J_{\lambda(l)+1}(\xi_{n\lambda(l)})}\frac{J_{\lambda(l\pm1)}(\xi_{n'\lambda(l\pm1)}u)}{J_{\lambda(l\pm1)+1}(\xi_{n'\lambda(l\pm1)})}udu.$$

We thus finally obtain

$$
\begin{aligned}
&\langle\psi_{n'l'p'}|Q_\pm|\psi_{nlp}\rangle \\
&\quad= \frac{2}{R}K^{\mp}_{n'nl}\delta_{l',l\mp1}\delta_{pp'} + \frac{4iqL}{\pi^2 R}K^{\mp}_{n'nl}\delta_{l',l\mp1}B_{pp'} \\
&\quad+ \frac{2}{R}\delta_{l',l\mp1}\sum_{n_1}\left(\frac{U_{n'l'p';n_1l'p}}{\epsilon_{n'l'p'}-\epsilon_{n_1l'p}}K^{\mp}_{n_1nl} + \frac{U_{n_1lp';nlp}}{\epsilon_{nlp}-\epsilon_{n_1lp'}}K^{\mp}_{n'n_1l}\right) \\
&\quad= \frac{2}{R}K^{\mp}_{n'nl}\delta_{l',l\mp1}\delta_{pp'} + \frac{4iqL}{\pi^2 R}K^{\mp}_{n'nl}\delta_{l',l\mp1}B_{pp'} \\
&\quad+ \frac{8ib}{L\pi^2R^3}\delta_{l',l\mp1}A_{pp'}\sum_{n_1}\left(\frac{l'I_{n'n_1l'}K^{\mp}_{n_1nl}}{\epsilon_{n'l'p'}-\epsilon_{n_1l'p}} + \frac{lI_{n_1nl}K^{\mp}_{n'n_1l}}{\epsilon_{nlp}-\epsilon_{n_1lp'}}\right) \\
&\quad= \frac{2}{R}K^{\mp}_{n'nl}\delta_{l',l\mp1}\delta_{pp'} \\
&\quad+ \frac{8i}{\pi^2R}A_{pp'}\delta_{l',l\mp1}\left[\frac{qLK^{\mp}_{n'nl}}{p^2-p'^2} + \frac{b}{LR^2}\sum_{n_1}\left(\frac{l'I_{n'n_1l'}K^{\mp}_{n_1nl}}{\epsilon_{n'l'p'}-\epsilon_{n_1l'p}} + \frac{lI_{n_1nl}K^{\mp}_{n'n_1l}}{\epsilon_{nlp}-\epsilon_{n_1lp'}}\right)\right].
\end{aligned}
$$

Circular dichroism

Consider intraband absorption of light by electrons of fixed energy generated through interband transitions by a linearly polarized pump. The energy of electrons can correspond to a nondegenerate state of zero angular momentum or a pair of doubly degenerate states of nonzero momentum. The rest of electronic states are assumed to be unoccupied, and the probe is assumed to be weak to prevent depletion of the excited state(s). Using Fermi's golden rule, it can then be shown that the difference of intraband transition rates due to left-hand and right-hand polarized light is given by

$$
\begin{aligned}
\Delta W &= W_L(\omega) - W_R(\omega) \\
&= \pi\left(\frac{A_0 e}{2mc}\right)^2\sum_{n'l'p'}\Big(\left|\langle\psi_{n'l'p'}|Q_-|\psi_{nlp}\rangle\right|^2 - \left|\langle\psi_{n'l'p'}|Q_+|\psi_{nlp}\rangle\right|^2 \\
&+ \left|\langle\psi_{n'l'p'}|Q_-|\psi_{n,-l,p}\rangle\right|^2 - \left|\langle\psi_{n'l'p'}|Q_+|\psi_{n,-l,p}\rangle\right|^2\Big)\,\delta(\omega-\omega_{n'l'p';nlp}).
\end{aligned}
$$

Here the summation extends over all the final states that are excited from a given initial state (nlp) by the probe of frequency ω.

It is convenient to consider separately transitions occurring with and without a change in the parity of the quantum number p. Using the selection rules to evaluate the summation over the final quantum number l', we obtain in the latter case

$$
\Delta W = \pi\left(\frac{A_0 e}{mcR}\right)^2\sum_{n'}\left(\left|K^+_{n'nl}\right|^2 - \left|K^-_{n'nl}\right|^2 + \left|K^+_{n'n,-l}\right|^2 - \left|K^-_{n'n,-l}\right|^2\right)\delta(\omega-\omega_{n'l'p;nlp}) = 0,
$$

where it has been taken into account that $K^{\pm}_{n'nl} = K^{\mp}_{n'n,-l}$. Hence, intraband transitions preserving the quantum number p are optically inactive.

If the parity of p changes, there are three kinds of intraband transitions:

(i) $l = 0 \to l' = \pm 1$;

(ii) $\pm l \to \pm l \pm 1$;

(iii) $\pm l \to \pm l \mp 1$.

For the transitions of the **first kind**, we obtain

$$\Delta W = \frac{\pi}{2}\left(\frac{A_0 e}{mc}\right)^2 \sum_{n'p'} \left(\left|\langle\psi_{n',1,p'}|Q_-|\psi_{n,0,p}\rangle\right|^2 - \left|\langle\psi_{n',-1,p'}|Q_+|\psi_{n,0,p}\rangle\right|^2\right)\delta\left(\omega-\omega_{n',1,p';n,0,p}\right)$$

$$= \frac{2^5}{\pi^3}\left(\frac{A_0 e}{mcR}\right)^2 \sum_{n'p'} A_{pp'}^2 \left[\left(\frac{qLK_{n'n0}^{+}}{p^2-p'^2} + \frac{b}{LR^2}\sum_{n_1}\frac{I_{n'n_1 1}K_{n_1 n0}^{+}}{\epsilon_{n',1,p'}-\epsilon_{n_1,1,p}}\right)^2 - \left(\frac{qLK_{n'n0}^{-}}{p^2-p'^2} - \frac{b}{LR^2}\sum_{n_1}\frac{I_{n'n_1,-1}K_{n_1 n0}^{-}}{\epsilon_{n',1,p'}-\epsilon_{n_1,1,p}}\right)^2\right]\delta\left(\omega-\omega_{n',1,p';n,0,p}\right)$$

$$= C_0 \sum_{n'p'n_1} \frac{A_{pp'}^2}{p^2-p'^2}\frac{I_{n'n_1,1}K_{n'n0}^{+}K_{n_1 n0}^{+}}{\epsilon_{n',1,p'}-\epsilon_{n_1,1,p}}\delta\left(\omega-\omega_{n',1,p';n,0,p}\right),$$

where

$$C_l = \left(1+\delta_{l,0}\right)\frac{2^6}{\pi^3}\left(\frac{A_0 e}{mc}\right)^2\frac{bq}{R^4}$$

and the identities $I_{n_1 n',l} = I_{n_1 n',-l}$ and $K_{n_1 n0}^{-} = K_{n_1 n0}^{+}$ have been used.

For the transitions of the **second kind**, we get

$$\Delta W = \frac{2^4}{\pi^3}\left(\frac{A_0 e}{mcR}\right)^2 \sum_{n'p'} A_{pp'}^2 \left\{\left[\frac{qLK_{n'nl}^{+}}{p^2-p'^2} + \frac{b}{LR^2}\sum_{n_1}\left(\frac{(l+1)I_{n'n_1,l+1}K_{n_1 nl}^{+}}{\epsilon_{n',l+1,p'}-\epsilon_{n_1,l+1,p}} + \frac{lI_{n_1 nl}K_{n'n_1 l}^{+}}{\epsilon_{nlp}-\epsilon_{n_1 lp'}}\right)\right]^2 - \left[\frac{qLK_{n'n,-l}^{-}}{p^2-p'^2} - \frac{b}{LR^2}\sum_{n_1}\left(\frac{(l+1)I_{n'n_1,-l-1}K_{n_1 n,-l}^{-}}{\epsilon_{n',l+1,p'}-\epsilon_{n_1,l+1,p}} + \frac{lI_{n_1 n,-l}K_{n'n_1,-l}^{-}}{\epsilon_{nlp}-\epsilon_{n_1 lp'}}\right)\right]^2\right\}\delta\left(\omega - \omega_{n',l+1,p';n,l,p}\right)$$

$$= C_l \sum_{n'p'n_1} \frac{A_{pp'}^2 K_{n'nl}^{+}}{p^2-p'^2}\left(\frac{(l+1)I_{n'n_1,l+1}K_{n_1 nl}^{+}}{\epsilon_{n',l+1,p'}-\epsilon_{n_1,l+1,p}} + \frac{lI_{n_1 nl}K_{n'n_1 l}^{+}}{\epsilon_{nlp}-\epsilon_{n_1 lp'}}\right)\delta\left(\omega - \omega_{n',l+1,p';n,l,p}\right).$$

And, finally, for the transitions of the **third kind**, we find

$$\Delta W = -C_l \sum_{n'p'n_1} \frac{A_{pp'}^2 K_{n'nl}^{-}}{p^2-p'^2}\left(\frac{(l-1)I_{n'n_1,l-1}K_{n_1 nl}^{-}}{\epsilon_{n',l-1,p'}-\epsilon_{n_1,l-1,p}} + \frac{lI_{n_1 nl}K_{n'n_1 l}^{-}}{\epsilon_{nlp}-\epsilon_{n_1 lp'}}\right)\delta\left(\omega-\omega_{n',l-1,p';n,l,p}\right).$$

It is seen that ΔW can be written in the general form as

$$\Delta W = \pm C_l \sum_{n'p'} \frac{A_{pp'}^2 K_{n'nl}^{\pm}}{p^2 - p'^2} F_{n'nl;pp'}^{\pm} \delta(\omega - \omega_{n',l\pm1,p';n,l,p}),$$

where

$$F_{n'nl;pp'}^{\pm} = \sum_{n_1} \left(\frac{(l \pm 1) I_{n'n_1,l\pm1} K_{n_1nl}^{\pm}}{\epsilon_{n',l\pm1,p'} - \epsilon_{n_1,l\pm1,p}} + \frac{l I_{n_1nl} K_{n'n_1l}^{\pm}}{\epsilon_{nlp} - \epsilon_{n_1lp'}} \right),$$

and where the upper sign corresponds to the transitions of the first and second kinds and the lower sign corresponds to transitions of the third kind.

Averaging over the spatial orientations of nanocrystals

To take into account the fact that nanocrystals are oriented randomly in real systems, we must average the obtained result over all possible nanocrystal orientations in space. To do this, we fix the orientation of a nanocrystal in space while assuming that $\mathbf{q} = q\mathbf{n}$ ($|\mathbf{n}| = 1$) and the vector potential of the probe is in the plane of orthogonal unit vectors $\boldsymbol{\eta}$ and $\boldsymbol{\xi}$. By employing our freedom in the choice of $\boldsymbol{\eta}$ and $\boldsymbol{\xi}$ associated with the circular polarization of light, we place $\boldsymbol{\eta}$ in the xy-plane and set the orientation of the reference frame $\{\boldsymbol{\eta}, \boldsymbol{\xi}, \mathbf{n}\}$ with respect to the frame (x, y, z) by a pair of angles. If ϑ is the angle between $\mathbf{n}$ and the z axis and γ is the angle between the projection of vector $\mathbf{n}$ to the xy-plane and the x axis (see Fig. 1), then

$$\boldsymbol{\eta} = (\sin\gamma, -\cos\gamma, 0),$$

$$\boldsymbol{\xi} = (\cos\gamma\cos\vartheta, \sin\gamma\cos\vartheta, -\sin\vartheta),$$

$$\mathbf{n} = (\cos\gamma\sin\vartheta, \sin\gamma\sin\vartheta, \cos\vartheta).$$

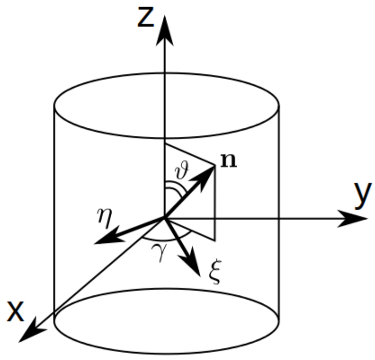

Figure 1: Relative orientation of nanocrystal coordinates (x, y, z) and orthogonal reper $\{\boldsymbol{\eta}, \boldsymbol{\xi}, \mathbf{n}\}$ determining the propagation direction ($\mathbf{n}$) and polarization of probe.

The interaction of electrons with left-hand and right-hand polarized light is described by the Hamiltonians

$$H_L = \frac{A_0 e}{2mc} e^{iq\mathbf{nr}} (i\boldsymbol{\eta} - \boldsymbol{\xi})\mathbf{p},$$

$$H_R = \frac{A_0 e}{2mc} e^{iq\mathbf{nr}} (i\boldsymbol{\eta} + \boldsymbol{\xi})\mathbf{p}.$$

Taking into account that

$$\mathbf{nr} = \frac{1}{2} r e^{i\varphi} e^{-i\gamma} \sin\vartheta + \frac{1}{2} r e^{-i\varphi} e^{i\gamma} \sin\vartheta + z\cos\vartheta,$$

$$(i\boldsymbol{\eta} - \boldsymbol{\xi})\mathbf{p} = -i\hbar \left(e^{i\gamma} \frac{1-\cos\vartheta}{2} S_+ - e^{-i\gamma} \frac{1+\cos\vartheta}{2} S_- + \sin\vartheta\, \partial_z \right),$$

and

$$(i\boldsymbol{\eta} + \boldsymbol{\xi})\mathbf{p} = -i\hbar \left(e^{i\gamma} \frac{1+\cos\vartheta}{2} S_+ - e^{i\gamma} \frac{1-\cos\vartheta}{2} S_- - \sin\vartheta\, \partial_z \right),$$

where

$$S_\pm = e^{\mp i\varphi} \left(\frac{\partial}{\partial r} \mp \frac{i}{r} \frac{\partial}{\partial \varphi} \right),$$

and expanding the exponential $e^{iq\mathbf{nr}}$ in Taylor's series, we obtain

$$H_L \approx -i \frac{A_0 e\hbar}{2mc} \left[1 + iq \left(\frac{1}{2} r e^{i\varphi} e^{-i\gamma} \sin\vartheta + \frac{1}{2} r e^{-i\varphi} e^{i\gamma} \sin\vartheta + z\cos\vartheta \right) \right] \left(e^{i\gamma} \frac{1-\cos\vartheta}{2} S_+ \right.$$
$$\left. - e^{-i\gamma} \frac{1+\cos\vartheta}{2} S_- + \sin\vartheta\, \partial_z \right),$$

$$H_R \approx -i \frac{A_0 e\hbar}{2mc} \left[1 + iq \left(\frac{1}{2} r e^{i\varphi} e^{-i\gamma} \sin\vartheta + \frac{1}{2} r e^{-i\varphi} e^{i\gamma} \sin\vartheta + z\cos\vartheta \right) \right] \left(e^{i\gamma} \frac{1+\cos\vartheta}{2} S_+ \right.$$
$$\left. - e^{-i\gamma} \frac{1-\cos\vartheta}{2} S_- - \sin\vartheta\, \partial_z \right).$$

By performing calculations similar to those described in Sections *Interaction of electrons with circularly polarized light* and *Circular dichroism*, one can show that not all terms in these Hamiltonians contribute to circular dichroism. In particular, the terms containing just operators ∂_z, $re^{i\varphi}\partial_z$, $re^{-i\varphi}\partial_z$, and $z\partial_z$ are irrelevant because they are symmetrically (up to a phase) contribute to Hamiltonians H_L and H_R whereas the selection rules for these operators are different. The terms with operators $re^{-i\varphi}S_+$, $re^{i\varphi}S_+$, $re^{-i\varphi}S_-$, and $re^{i\varphi}S_-$ do not contribute to circular dichroism either, as they cancel in pairs due to the degeneracy of electronic states in angular momentum projections.

By discarding the vanishing terms, we get

$$H_L \propto (1 + iqz\cos\vartheta) \left(e^{i\gamma} \frac{1-\cos\vartheta}{2} S_+ - e^{-i\gamma} \frac{1+\cos\vartheta}{2} S_- \right),$$

$$H_R \propto (1 + iqz\cos\vartheta) \left(e^{i\gamma} \frac{1+\cos\vartheta}{2} S_+ - e^{-i\gamma} \frac{1-\cos\vartheta}{2} S_- \right).$$

Since circular dichroism depends on the modules of the matrix elements of operators H_L and H_R, and the selection rules for operators S_+ and S_- are different, the phase factors $e^{i\gamma}$ and $e^{-i\gamma}$ can be omitted. Circular dichroism can then be calculated similar to how it was done before to obtain

$$\Delta W \propto \cos\vartheta \sum_{n'l'p'} \left[\left(\frac{1+\cos\vartheta}{2}\right)^2 \left|\langle \psi_{n'l'p'} | Q_- | \psi_{nlp} \rangle\right|^2 - \left(\frac{1-\cos\vartheta}{2}\right)^2 \left|\langle \psi_{n'l'p'} | Q_- | \psi_{nlp} \rangle\right|^2 \right.$$
$$- \left(\frac{1+\cos\vartheta}{2}\right)^2 \left|\langle \psi_{n'l'p'} | Q_+ | \psi_{nlp} \rangle\right|^2 + \left(\frac{1-\cos\vartheta}{2}\right)^2 \left|\langle \psi_{n'l'p'} | Q_+ | \psi_{nlp} \rangle\right|^2$$
$$+ \left(\frac{1+\cos\vartheta}{2}\right)^2 \left|\langle \psi_{n'l'p'} | Q_- | \psi_{n,-l,p} \rangle\right|^2 - \left(\frac{1-\cos\vartheta}{2}\right)^2 \left|\langle \psi_{n'l'p'} | Q_- | \psi_{n,-l,p} \rangle\right|^2$$
$$- \left(\frac{1+\cos\vartheta}{2}\right)^2 \left|\langle \psi_{n'l'p'} | Q_+ | \psi_{n,-l,p} \rangle\right|^2$$
$$\left. + \left(\frac{1-\cos\vartheta}{2}\right)^2 \left|\langle \psi_{n'l'p'} | Q_+ | \psi_{n,-l,p} \rangle\right|^2 \right]$$
$$\propto \left[\left(\frac{1+\cos\vartheta}{2}\right)^2 - \left(\frac{1-\cos\vartheta}{2}\right)^2 \right] \cos\vartheta = \cos^2\vartheta.$$

Then averaging over all possible orientations of the nanocrystal results in the factor

$$\frac{1}{\pi}\int_0^\pi \cos^2\vartheta \, d\vartheta = \frac{1}{2}.$$

Long nanocrystals

For those nanocrystals whose lengths are comparable to the probe's wavelength, the complete exponent e^{iqz} must be used in the electron–photon Hamiltonian. In this case, we find

$$\left\langle \psi_{n'l'p'}^{(0)} \middle| e^{iqz \mp i\varphi} \left(\frac{\partial}{\partial r} \mp \frac{i}{r}\frac{\partial}{\partial \varphi}\right) \middle| \psi_{nlp}^{(0)} \right\rangle = \frac{4}{\pi R} K_{n'nl}^{\mp} \delta_{l',l\mp 1} G_{pp'},$$

where

$$G_{pp'} = \int_{-\frac{\pi}{2}}^{\frac{\pi}{2}} du \, e^{i\sigma u} \sin(p'u)\sin(pu) = \int_{-\frac{\pi}{2}}^{\frac{\pi}{2}} du \, e^{i\sigma u} \cos(p'u)\cos(pu)$$
$$= \int_{-\frac{\pi}{2}}^{\frac{\pi}{2}} du \, e^{i\sigma u} \sin(p'u)\cos(pu)$$
$$= -\frac{4pp'\sigma}{[(p-p')^2 - \sigma^2][(p+p')^2 - \sigma^2]} \left[\frac{(-1)^p + (-1)^{p'}}{2} \cos\left(\frac{p+p'}{2}\pi\right) \sin\left(\frac{\pi\sigma}{2}\right) \right.$$
$$\left. + i \sin\left(\frac{p+p'}{2}\pi\right) \cos\left(\frac{\pi\sigma}{2}\right) \right]$$

and $\sigma = qL/\pi$.

It is easy to show that for $\sigma \ll 1$

$$G_{pp'} \approx \frac{\pi}{2}\delta_{pp'} + i\sigma B_{pp'}.$$

Thus, in the general case of long nanocrystals, we have

$$\langle\psi_{n'l'p'}|Q_\pm|\psi_{nlp}\rangle$$
$$= \frac{4}{\pi R}K^{\mp}_{n'nl}\delta_{l',l\mp1}G_{pp'}$$
$$+\frac{4}{\pi R}\delta_{l',l\mp1}\sum_{n_1p_1}\left(\frac{U_{n'l'p';n_1l'p_1}}{\epsilon_{n'l'p'}-\epsilon_{n_1l'p_1}}G_{p_1p}K^{\mp}_{n_1nl}+\frac{U_{n_1lp_1;nlp}}{\epsilon_{nlp}-\epsilon_{n_1lp_1}}G_{p'p_1}K^{\mp}_{n'n_1l}\right)$$
$$= \frac{4}{\pi R}K^{\mp}_{n'nl}\delta_{l',l\mp1}G_{pp'}$$
$$+\frac{16ib}{L(\pi R)^3}\delta_{l',l\mp1}\sum_{n_1p_1}\left(\frac{l'I_{n'n_1l'}A_{p_1p'}K^{\mp}_{n_1nl}G_{p_1p}}{\epsilon_{n'l'p'}-\epsilon_{n_1l'p_1}}+\frac{lI_{n_1nl}A_{pp_1}K^{\mp}_{n'n_1l}G_{p'p_1}}{\epsilon_{nlp}-\epsilon_{n_1lp_1}}\right)$$
$$= \frac{4}{\pi R}\delta_{l',l\mp1}\Bigg[K^{\mp}_{n'nl}G_{pp'}$$
$$+\frac{4ib}{L(\pi R)^2}\sum_{n_1p_1}\left(\frac{l'I_{n'n_1l'}A_{p_1p'}K^{\mp}_{n_1nl}G_{p_1p}}{\epsilon_{n'l'p'}-\epsilon_{n_1l'p_1}}+\frac{lI_{n_1nl}A_{pp_1}K^{\mp}_{n'n_1l}G_{p'p_1}}{\epsilon_{nlp}-\epsilon_{n_1lp_1}}\right)\Bigg].$$

As before, ΔW can be written as

$$\Delta W = \pm\left(1+\delta_{l,0}\right)\frac{2^6}{\pi^3}\left(\frac{A_0 e}{mc}\right)^2\frac{b}{LR^4}\sum_{n'p'}K^{\pm}_{n'nl}\mathrm{Im}\left(G_{pp'}M^{\pm*}_{n'nl;pp'}\right)\delta\left(\omega-\omega_{n',l\pm1,p';n,l,p}\right),$$

where

$$M^{\pm}_{n'nl;pp'} = \sum_{n_1p_1}\left(\frac{(l\pm1)I_{n'n_1,l\pm1}A_{p_1p'}K^{\pm}_{n_1nl}G_{p_1p}}{\epsilon_{n',l\pm1,p'}-\epsilon_{n_1,l\pm1,p_1}}+\frac{lI_{n_1nl}A_{pp_1}K^{\pm}_{n'n_1l}G_{p'p_1}}{\epsilon_{nlp}-\epsilon_{n_1lp_1}}\right).$$
